# The impact of antihypertensive treatment of mild to moderate hypertension during pregnancy on maternal and neonatal outcomes: An updated meta‐analysis of randomized controlled trials

**DOI:** 10.1002/clc.24013

**Published:** 2023-03-28

**Authors:** Armin Attar, Alireza Hosseinpour, Mana Moghadami

**Affiliations:** ^1^ Department of Cardiovascular Medicine, School of Medicine Shiraz University of Medical Sciences Shiraz Iran; ^2^ Clinical Education Research Center, Department of Medicine Shiraz University of Medical Sciences Shiraz Iran

**Keywords:** antihypertensive treatment, chronic hypertension, fetal outcomes, gestational hypertension, maternal outcomes

## Abstract

Currently, there is controversy regarding the treatment of pregnant patients with mild hypertension (blood pressure 140–159/90–109 mm Hg). While guidelines do not recommend this treatment, results from recent clinical trials are supportive of the treatment. This meta‐analysis aimed to clarify if active treatment of mild hypertension during pregnancy results in better maternal and fetal outcomes. All of the potentially eligible randomized controlled trials were retrieved through a systematic database search investigating the impact of pharmacological treatment in mild hypertensive patients on maternal, fetal, and neonatal outcomes. Relative risk (RR) and 95% confidence interval (CI) were calculated using a random‐effects model. Data from 12 trials comprising 4461 pregnant women diagnosed with mild to moderate hypertension (2395 in the intervention group and 2066 in the control group) were extracted for quantitative synthesis. Antihypertensive treatment was associated with better outcomes in seven out of the 19 analyzed outcomes: Severe hypertension (RR = 0.53; 95% CI = [0.38;0.75]), preeclampsia (RR = 0.71; 95% CI = [0.54; 0.93]), placental abruption (RR = 0.48; 95% CI = [0.26; 0.87]), changes in electrocardiogram (RR = 0.43; 95% CI = [0.25; 0.72]), renal impairment (RR = 0.42; 95% CI = [0.34; 0.51]), pulmonary edema (RR = 0.46; 95% CI = [0.25; 0.84]), and neonatal mortality (RR = 0.72; 95% CI = [0.57; 0.92]). The primary safety outcome of small for gestational age was not different between the treatment group and the control group (RR = 1.12; 95% CI = [0.80; 1.57]). The results of this meta‐analysis are in favor of the beneficial impact of pharmacological treatment of mild hypertension on both maternal and neonatal outcomes and without significant adverse events for the fetus.

## Graphical abstract

The potential beneficial or harmful effects of antihypertensive treatment of pregnant women with mild to moderate hypertension on maternal, fetal, and neonatal outcomes were investigated. Pharmacological treatment of mild to moderate hypertension during pregnancy was associated with lower risks of adverse maternal outcomes including severe hypertension, preeclampsia, and placental abruption. It was also considered safe for the fetus. Since antihypertensive medications appear to have beneficial impacts on pregnancy outcomes in mild hypertension, the target of blood pressure may be lowered to less than 140/90 mm Hg during pregnancy in hypertensive patients.

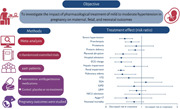

## 1 INTRODUCTION

Hypertensive disorders of pregnancy have been associated with a significantly higher risk of adverse pregnancy outcomes including fetal and neonatal death and small for gestational age (SGA).^1^, ^2^, ^3^ Although guidelines have reached a consensus on treating cases with severe hypertension (blood pressure ≥ 160/110 mm Hg),^4^ uncertainty still remains regarding the decision to treat patients with mild to moderate hypertension. Previously, the results of the CHIPS trial^5^ showed that tight control of gestational and chronic hypertension during pregnancy (target diastolic blood pressure [DBP] < 85 mm Hg) could not lower the risk of adverse maternal and perinatal outcomes when compared to the less‐tight control group (target DBP of 100 mm Hg). The recently published CHAP trial assigned 2408 patients with mild chronic hypertension during pregnancy to either antihypertensive treatment (first‐line drugs) with a blood pressure goal of <140/90 mm Hg or no treatment for hypertension unless the blood pressure reached severe hypertension (control group). They demonstrated that the risk of the primary composite outcome of preeclampsia with severe features, preterm birth, placental abruption, or perinatal death was significantly lower in patients treated with antihypertensive medications. Also, the safety outcome which was SGA below the 10th percentile did not differ between the two groups proposing better outcomes of pregnancy without any observed harm in mild hypertensive patients treated with medications compared to no treatment.^6^ The results of this trial suggest the beneficence of pharmacologic treatment of mild chronic hypertension during pregnancy to a blood pressure goal of below 140/90 mm Hg as supported by the latest statement made by the Society for Maternal‐Fetal Medicine (SMFM).^7^ Herein, we presented a meta‐analysis of the impact of pharmacological treatment in mild to moderate hypertension during pregnancy on maternal, neonatal, and fetal outcomes with randomized controlled trials (RCTs) being stratified by their type of hypertension (chronic and gestational or pregnancy‐induced).

## 2 METHODS

This is a systematic review and meta‐analysis aiming to investigate the potential impact of antihypertensive treatment in patients with mild chronic or gestational hypertension on pregnancy outcomes. We followed and reported this study based on the recommendations made by the Preferred Reporting Items for Systematic Review and Meta‐Analysis (PRISMA) 2020 Checklist.^8^

### 2.1 Information sources and search strategy

Relevant keywords related to hypertension and outcomes of pregnancy in patients receiving antihypertensive treatment in combination with the medical subject heading terms were used in a systematic search through PubMed, Embase, and Scopus from the database inception to August 2022 with no specific filters. The detailed search terms in each of the databases are provided in the Supporting Information: Material. Additionally, the bibliographies of the previous meta‐analyses were screened for further potentially eligible studies.

### 2.2 Selection process and eligibility criteria

All the records found in database searching were retrieved for further screening. After the removal of the duplicate records, two reviewers (A. H. and M. M.) screened the titles and abstracts using the Rayyan web‐based tool^9^ and selected the potentially eligible studies based on the eligibility criteria. The potentially relevant records were retrieved for full‐text screening and citation searching for further relevant studies. The eligible studies were rechecked by a third investigator (A. A.) for confirmation.

The inclusion criteria comprised all of the RCTs of pregnant women diagnosed with either mild to moderate (defined as 140 mm Hg ≤ systolic blood pressure [SBP] < 160 mm Hg or 90 mm Hg ≤ SBP < 110 mm Hg) chronic (present before pregnancy or diagnosed before 20 weeks of pregnancy) or pregnancy‐induced (gestational) hypertension (diagnosis made after 20 weeks of gestation) with no proteinuria and target organ diseases comparing maternal and fetal outcomes in experimental (receiving antihypertensive medications) versus control group (receiving either placebo or no treatment). The exclusion criteria were as follows:

1. All the observational studies, case reports, case series, case‐control studies, reviews, animal studies, abstracts that an original article had not been published in the literature, and meta‐analyses.
2. Studies that did not include a control group of hypertensive patients or compared the intervention group to a group of normal pregnant women.
3. Studies involving patients with preeclampsia or hypertensive patients with proteinuria.
4. Studies include pregnant patients with SBP ≥ 160 mm Hg or DBP ≥ 110 mm Hg.
5. Studies or groups that were designed to compare aspirin use during pregnancy. Since we wanted to investigate the impact of only antihypertensive medications on pregnancy outcomes, aspirin use may be a confounding factor given the fact that low‐dose aspirin has been shown to be effective in the prevention of adverse maternal and perinatal outcomes such as preeclampsia, preterm birth, and perinatal mortality^10^ and the mentioned variables were among our outcomes of interest.

### 2.3 Outcomes of interest, data collection process, and risk of bias

The endpoints of this meta‐analysis included all the maternal, fetal, and neonatal outcomes mentioned in the trials such as severe hypertension, superimposed preeclampsia, placental abruption, and low birth weight (LBW). The definition of each of the study variables is presented in Supporting Information: Table S1. For the safety outcome, our primary outcome was the incidence of SGA. The data were collected by two investigators (A. H. and M. M.) independently and rechecked after the process with discrepancies being resolved through discussion. We extracted data on the year of publication, country of the trial that took place, study design, type of hypertension (chronic, gestation, and mixed), the sample size of the experimental and control groups, maternal outcomes (including severe hypertension, preeclampsia, preterm labor, premature rupture of membrane, placental abruption, hospital admission, heart failure, stroke, venous thromboembolism, myocardial infarction, electrocardiogram [ECG] changes, hepatic and renal impairment, pulmonary edema, proteinuria, and maternal mortality) and fetal and neonatal outcomes (fetal growth restriction, SGA, intrauterine fetal demise (IUFD), LBW, very LBW, admission to neonatal intensive care unit [NICU], Apgar score <7 at 5 min, and neonatal mortality).

For the qualitative assessment of the eligible RCTs included in this study, we used the Cochrane Collaboration's tool for risk of bias assessment.^11^ This tool assesses the risk of bias in five categories of selection, performance, detection, attrition, and reporting bias. The risk of bias in each category was rated by an author (A. H.). The quality of the included studies was visualized in a figure using Review Manager Software.

### 2.4 Statistical analysis

We investigated the impact of pharmacological treatment on maternal, neonatal, and fetal outcomes in mild to moderate hypertensive patients during pregnancy. Thus, we compared the effect of treatment with a control group using the random‐effects model and generated a risk ratio (RR) and its 95% confidence interval (CI). Antihypertensive treatment was associated with better outcomes if the RR < 1 and in the case of RR > 1 treatment was considered to increase the RR of that specific outcome. If the data on an outcome were repeated in two or more studies, it was included for analysis. Meta‐analysis of proportions was conducted to estimate the overall proportions of all the outcomes included for analysis using the inverse variance method. The results of this meta‐analysis were visualized by generating forest plots. A subgroup analysis was performed for all the analyses based on the type of hypertension (chronic, gestational, or both) included in each of the trials. All the analyses were performed using Mantel–Haenszel method. The level of heterogeneity between studies was quantified using *I* ^2^ statistics and the studies were categorized as considerable, substantial, and moderate heterogeneity if *I* ^2^ > 75%, 50%–75%, and 30%–50%, respectively. For the qualitative assessment of publication bias, we generated funnel plots and for the quantitative assessment, we calculated Peter's test *p* value if there were ≥10 studies included in the analysis. In each of the analyses, if the overall pooled estimate of RR did not cross the line of 1, it was considered to be statistically significant. Sensitivity analysis was conducted by deleting the outlier studies and reanalyzing the remaining ones. The data were analyzed using RStudio software version 1.3.959 with “meta” and “dmetar” packages being used.

## 3 RESULTS

### 3.1 Search results and description of studies

**Figure 1:**
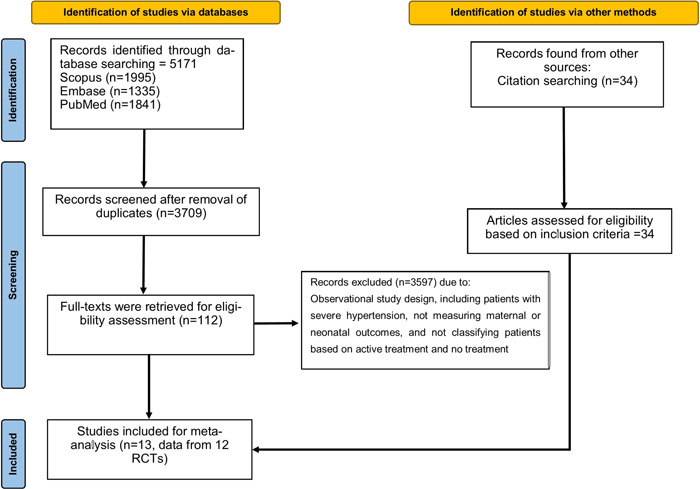
PRISMA flowchart of the process of study inclusion. PRISMA, Preferred Reporting Items for Systematic Review and Meta‐analysis; RCT, randomized controlled trials.

The primary search through databases yielded a total of 5171 records across PubMed, Scopus, and Embase. Following the removal of 1462 duplicate studies, titles, and abstracts of 3709 articles were retrieved for checking the potential eligibility by applying the exclusion criteria. After discarding the irrelevant papers (*n* = 3597), we carefully evaluated the full text of the remaining 112 studies and finally, 13 studies^6^, ^12^, ^13^, ^14^, ^15^, ^16^, ^17^, ^18^, ^19^, ^20^, ^21^, ^22^, ^23^ from 12 RCTs including 4461 pregnant patients diagnosed with mild to moderate hypertension (2395 participants in the experimental group and 2066 in the control group) were chosen for inclusion in the analyses. The data were extracted from six trials^6^, ^12^, ^18^, ^19^, ^21^, ^22^ including patients with chronic hypertension, three with gestational hypertension,^13^, ^14^, ^15^ and three with both types of hypertensive patients.^16^, ^17^, ^23^ The included studies varied in the sample size with the smallest trial^22^ including 58 pregnant patients and the largest one^6^ comprising 2408 patients. PRISMA flowchart of the study is provided in Figure 1. Table 1 summarizes the details of the included trials.

**Table 1:** Characteristics of the included studies.

Study	Country	Year of publication	Type of hypertension	Sample size		Mean age		Treatment goal	Intervention	Definition of hypertension
				Inv	Ctrl	Inv	Ctrl			
Arias and Zamora^22^	United States	1979	Chronic	29	29	32.3 ± 1.1	30.8 ± 0.9	DBP < 90 mm Hg	Methyldopa 750 mg/day (initial), hydralazine 75 mg/day (initial), hydrochlorothiazide 50 mg/day (regimens: methyldopa and thiazide or hydralazine and thiazide, or methyldopa, hydralazine, and thiazide)	Blood pressure ≥ 140/90 mm Hg and DBP < 100 mm Hg before pregnancy or 20 weeks of gestation
Bott‐Kanner et al.^23^	Israel	1992	Mixed	30	30	30.4 ± 5.3	31.4 ± 5.4	DBP < 85 mm Hg	The initial dose of one tablet of BID and hydralazine added at increasing doses of up to 200 mg/day	DBP of 85–99 mm Hg on two examinations at least 12 h apart
Tita et al.^6^	United States	2022	Chronic	1208	1200	32.3 ± 5.6	32.3 ± 5.8	BP < 140/90 mm Hg	First‐line (labetalol, extended‐release nifedipine, amlodipine, or methyldopa) and second medication (nifedipine or labetalol)	Blood pressure ≥ 140/90 mm Hg on at least two occasions and at least 4 h apart before 20 weeks of gestation
Vigil‐De Garcia et al.^19^	Panama	2014	Chronic	41	19	34.3 ± 4.8	33.9 ± 4.2		Furosemide group: 20 mg furosemide per day, amlodipine group: 5 mg amlodipine per day until the 37th week of gestation	SBP of 140–159 mm Hg and DBP of 90–109 mm Hg before 20 weeks of gestation or pregnancy
Hogstedt et al.^16^	Sweden	1985	Mixed	82	79	29.2 ± 5.3	29.2 ± 4.8	DBP <90 mm Hg	Metoprolol 50 mg and hydralazine 25 mg twice daily (initial)	DBP of 90–110 mm Hg on two or more measurements
Molvi et al.^13^	India	2012	Gestational	99	50	25.4 ± 3.3	24.9 ± 2.6	BP <140/90 but >170/110 mm Hg	Labetalol group: 100 mg twice daily (initial), methyldopa group: 250 mg twice daily (initial)	SBP of 140–159 mm Hg and DBP of 90–109 mm Hg on two occasions 6 h apart in the office after 20 weeks of gestation
Parazzini et al.^17^	Italy	1998	Mixed	132	129	31.3 ± 5.6	31.2 ± 5.6	–	Slow‐release nifedipine initial dose of 10 mg twice daily	DBP of 90–110 mm Hg on two occasions 4 h apart between 12 and 34 weeks of gestation
Pickles et al.^15^	United Kingdom	1989	Gestational	70	74	27.5 ± 5.8	24.7 ± 4.8	–	Labetalol three tablets per day for the initial dose	SBP of 140–160 mm Hg and DBP of 90–105 mm Hg after 15 min of rest 24 h apart between 20 and 38 weeks of gestation
Plouin et al.^14^	France	1990	Gestational	78	76	26.1 ± 7	25.9 ± 6	DBP < 86 mm Hg	Starting with oxprenolol 160 mg twice orally per day then advancing to 320 mg of oxprenolol plus 100 mg hydralazine daily	DBP higher than 84 mm Hg on two occasions after 20 weeks of gestation in the sitting position after 5 min of rest
Salama et al.^18^	Egypt	2019	Chronic	326	164	20–40	20–40	DBP < 100 and SBP < 160 mm Hg	Methyldopa group: 1–2 g per day in divided doses and Nifedipine group: 20–40 mg/day in divided doses	SBP of 140–159 mm Hg and DBP of 90–109 mm Hg between 6 and 10 weeks of pregnancy without features of end‐organ damage
Sibai et al.^21^	United States	1990	Chronic	173	90	30.9 ± 0.7	29 ± 0.6	SBP < 140 and DBP < 90 mm Hg	Methyldopa 750 mg/day and increased to a maximum dose of 2400 mg/day, if the blood pressure control was inadequate, hydralazine 300 mg/day was added.	History of mild chronic hypertension before pregnancy
Xiang et al.^12^	China	2020	Chronic	127	126	19–39	19–39	DBP < 100–105 and SBP < 160 mm Hg	Labetalol (dosage not specified)	SBP of 140–159 mm Hg and DBP of 90–109 mm Hg between 6 and 10 weeks of pregnancy without medication and target organ damage

Abbreviations: BP, blood pressure; Ctrl, control group; DBP, diastolic blood pressure; Inv, intervention group; SBP, systolic blood pressure.

### 3.2 Quality assessment, publication bias, and sensitivity analysis

Almost all the studies were at low risk of bias due to using a clear method for random sequence generation including randomization in blocks of six with serial numbers,^23^ labeled allocations cards numbered 1–150,^13^ a list generated by a computer,^17^ a list of random numbers,^15^ stratified blocks of 10 in 2 centers,^14^ computer‐generated simple random tables,^18^ random numbers generated by the computer,^21^ web‐based randomization program with variable block sizes of 2, 4, and 6,^6^ block sizes of 6 generated with the computer,^19^ and simple random table.^12^ Allocation concealment was at unclear or high risk of bias in half of the included studies.^12^, ^13^, ^16^, ^17^, ^21^, ^22^ Three studies were open‐label trials and hence were rated as high risk for performance bias.^6^, ^16^, ^19^ Blinding of outcome assessors was not determined in the majority of the trials, thus the detection bias was rated as unclear.^12^, ^13^, ^14^, ^15^, ^16^, ^17^, ^19^, ^20^, ^21^, ^22^, ^23^ The risk of bias summary and graph are displayed in Supporting Information: Figure S1.

Publication bias was assessed to explore the potential small study effects in case of 10 or more studies were available. Visual inspection of the funnel plot for SGA showed a relatively symmetrical distribution of the studies (Supporting Information: Figure S2) (*p* = .8). For sensitivity analysis, we removed each of the studies one at a time for primary outcomes to see their impact on the summary of results, and no significant change was observed for any of the outcomes (Supporting Information: Figures S3–5).

### 3.3 Maternal outcomes

**Figure 2:**
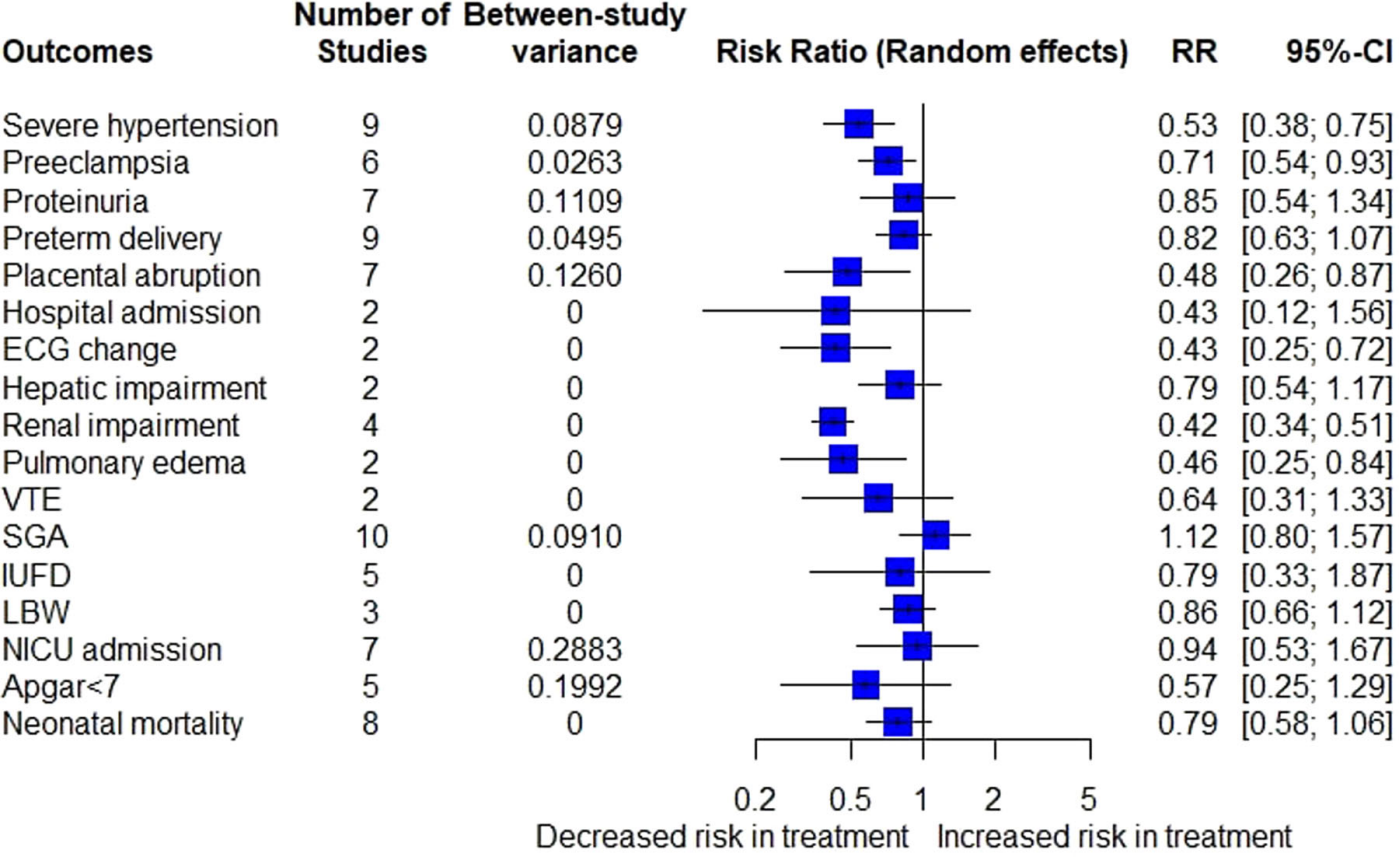
Pooled treatment effect estimates of antihypertensive treatment in mild hypertension on pregnancy outcomes. 95% CI, 95% confidence interval; ECG, electrocardiogram; IUFD, intrauterine fetal demise; LBW, low birth weight; NICU, neonatal intensive care unit; RR, relative risk; SGA, small for gestational age; VTE, venous thromboembolism.

**Figure 3:**
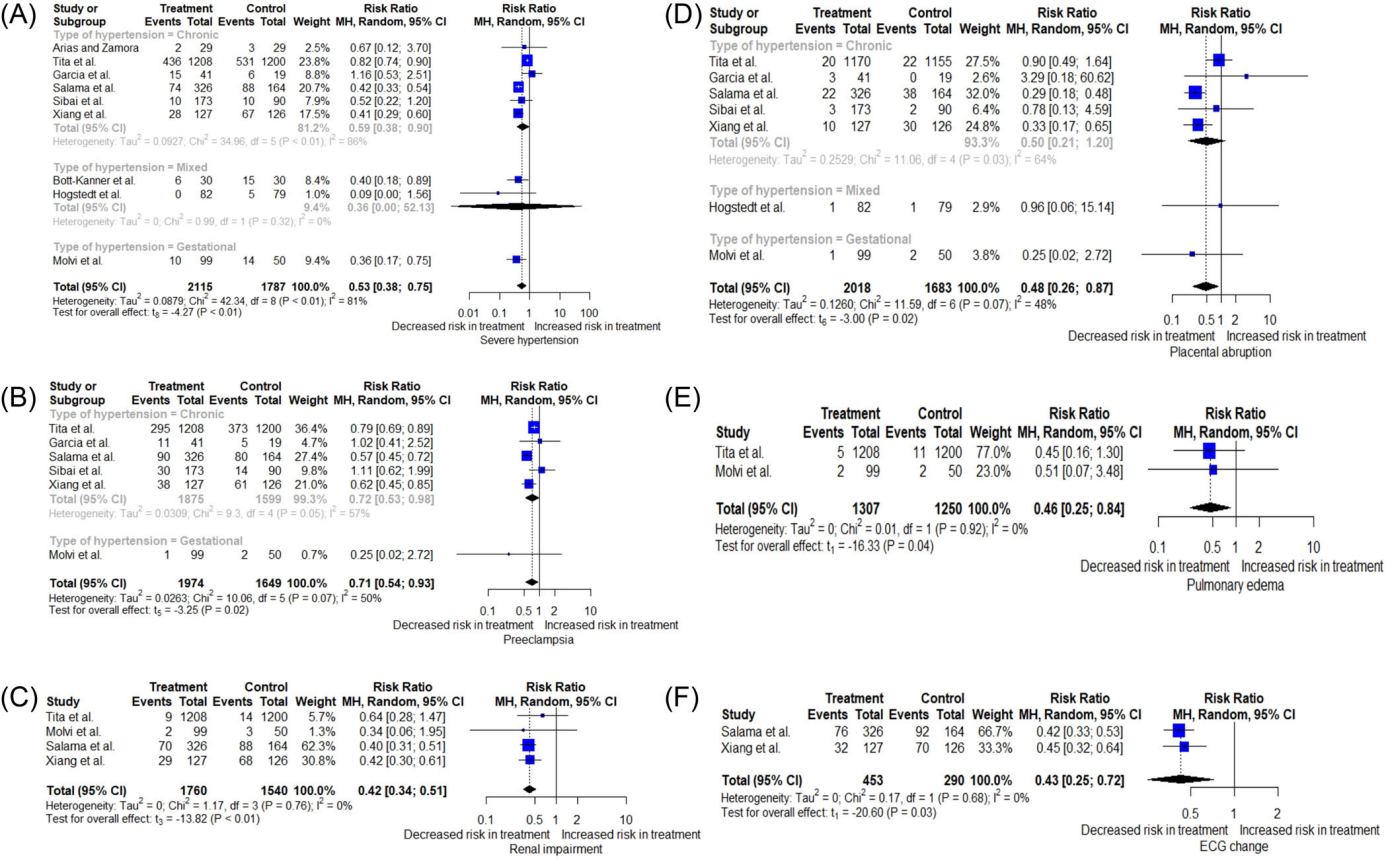
Forest plot displaying the impact of antihypertensive medications in mild hypertension during pregnancy on (A) severe hypertension, (B) preeclampsia, (C) renal impairment, (D) placental abruption, (E) pulmonary edema, and (F) ECG change based on the type of hypertension included in each trial. 95% CI, 95% confidence interval; ECG, electrocardiogram; MH, Mantel–Haenszel.

The impact of antihypertensive therapy on maternal outcomes in pregnant patients diagnosed with mild to moderate hypertension was investigated (Figure 2). Antihypertensive therapy of mild to moderate hypertension was associated with lower risks of developing severe hypertension (16% vs. 29%; RR = 0.53; 95% CI = [0.38; 0.75]) (Figure 3A), preeclampsia (24% vs. 27%; RR = 0.71; 95% CI = [0.54; 0.93]) (Figure 3B), placental abruption (3% vs. 5%; RR = 0.48; 95% CI = [0.26; 0.87]) (Figure 3D), changes in ECG (24% vs. 56%; RR = 0.43; 95% CI = [0.25; 0.72]) (Figure 3F), renal impairment (6% vs. 15%; RR = 0.42; 95% CI = [0.34; 0.51]) (Figure 3C), and pulmonary edema (1% vs. 2%; RR = 0.46; 95% CI = [0.25; 0.84]) (Figure 3E) compared to the control group receiving either no treatment or placebo. The pooled analysis showed that antihypertensive medications had no statistically significant impact on proteinuria (16% vs. 18%; RR = 0.85; 95% CI = [0.54; 1.34]), preterm delivery (20% vs. 25%; RR = 0.82; 95% CI = [0.63; 1.07]), hospital admission (19% vs. 45%; RR = 0.43; 95% CI = [0.12; 1.56]), maternal mortality (0% vs. 0%; RR = 0.34; 95% CI = [0.00; 241.74]), hepatic impairment (23% vs. 29%; RR = 0.79; 95% CI = [0.54; 1.17]), heart failure (0% vs. 0%; RR = 1.54; 95% CI = [0.00; 693.85]), and venous thromboembolism (2% vs. 4%; RR = 0.64; 95% CI = [0.31; 1.33]) when compared to the control group receiving no treatment or placebo for nonsevere hypertension (Figure 2) (the pooled estimates of maternal mortality and heart failure is not presented in Figure 2 due to having very wide CIs and their forest plots are presented in the Supporting Information: Material).

### 3.4 Fetal and neonatal outcomes

Pooled analysis displayed no statistically significant decrease in the risk of SGA (14% vs. 13%; RR = 0.1.12; 95% CI = [0.80; 1.57]), IUFD (3% vs. 3%; RR = 0.79; 95% CI = [0.33; 1.87]), LBW (23% vs. 24%; RR = 0.86; 95% CI = [0.66; 1.12]), NICU admission (18% vs. 19%; RR = 0.94; 95% CI = [0.53; 1.67]), Apgar <7 (6% vs. 7%; RR = 0.57; 95% CI = [0.25; 1.29]), and neonatal mortality (3% vs. 4%; RR = 0.79; 95% CI = [0.58; 1.06]) associated with the use of antihypertensive drugs (Figure 2). After excluding a study with a high chance of bias,^12^ the analysis revealed a significant decrease in the risk of neonatal mortality in cases treated with medications compared to the control group (RR = 0.72; 95% CI = [0.57; 0.92]) (Supporting Information: Figure S6).

### 3.5 Subgroup analysis

**Figure 4:**
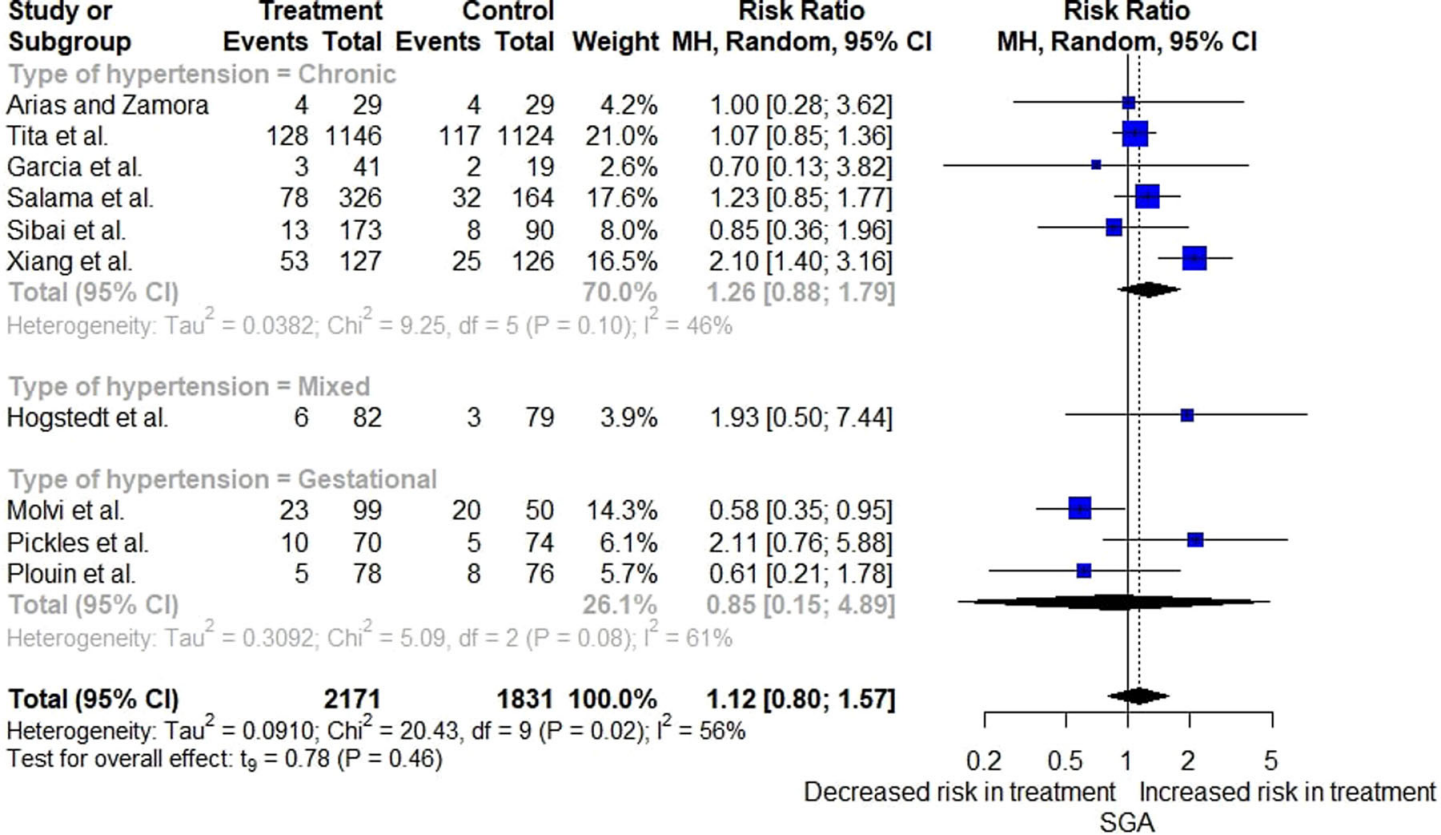
Forest plot displaying the impact of antihypertensive medications in mild hypertension during pregnancy on small for gestational age (SGA) based on the type of hypertension included in each trial. 95% CI, 95% confidence interval; MH, Mantel–Haenszel.

Results of each of the analyses were stratified by the type of hypertension enrolled in each trial (chronic, gestational, or mixed) (Supporting Information: Figures S7–18). A trend toward benefit was observed for both types of hypertension in decreasing the risk of developing severe hypertension in the active treatment group compared to the control group (RR = 0.59; 95% CI = [0.38; 0.90] and RR = 0.36; 95% CI = [0.17; 0.75] for chronic and gestational hypertension, respectively) with a considerable level of heterogeneity (*I* ^2^ = 86%) (Figure 3A). Also, antihypertensive therapy resulted in decreased risk of developing preeclampsia (RR = 0.71; 95% CI = [0.54; 0.93]; *I* ^2^ = 50%) (Figure 3B). For the primary safety outcome, there was no significant difference in the incidence of SGA between the two groups amongst pregnant patients with mild chronic and gestational hypertension (RR = 1.26; 95% CI = [0.88; 1.79] and RR = 0.85; 95% CI = [0.15; 4.89], respectively) (*I* ^2^ = 56%) (Figure 4). The pooled analysis showed that antihypertensive therapy could significantly lower the risk of preterm delivery in patients with chronic hypertension (RR = 0.86; 95% CI = [0.78; 0.95]), whereas this result was not achieved in patients with gestational hypertension (RR = 0.43; 95% CI = [0.10; 1.85]) (Supporting Information: Figure S8).

## 4 DISCUSSION

The present meta‐analysis including 4461 participants investigated the potential effect of pharmacological therapy on the maternal and fetal outcomes of patients with mild to moderate hypertension during pregnancy. Herein, we focused only on the studies comparing active treatment versus no treatment or placebo in targeting mild to moderate hypertension during pregnancy which has been a matter of a long‐standing dispute. Overall, it can be demonstrated that antihypertensive treatment contributed to a lower risk of some of the maternal outcomes such as severe hypertension (RR = 0.53 [0.38; 0.75]), preeclampsia (RR = 0.71 [0.54; 0.93]), placental abruption (RR = 0.48 [0.26; 0.87]), and renal impairment (RR = 0.42 [0.34; 0.51]). Also, the antihypertensive treatment showed a significant decrease in the chances of neonatal mortality following the exclusion of a study with a high risk of bias (RR = 0.72 [0.57; 0.92]). Notably, active treatment of mild hypertension did not differ from the control group not receiving medication in terms of any of the safety outcomes in our analysis.

The results of this meta‐analysis are in accordance with the findings of the recently published CHAP trial. The CHAP study, which was a multicenter randomized open‐label trial, examined the effect of treatment in pregnant women with mild chronic hypertension randomized before 23 weeks of gestation and the target blood pressure control was less than 140/90 mm Hg. The incidence of their primary endpoint which was a composite of preeclampsia, preterm birth, placental abruption, and neonatal mortality, was significantly lower in patients receiving treatment compared to the control group (30.2% vs. 37%; RR [95% CI] = 0.82 [0.74; 0.92]). Based on a prespecified subgroup analysis, the effect of treatment on the primary outcome was more pronounced in the subgroup of previously diagnosed hypertensive patients who were already on medications compared to newly diagnosed patients and previously diagnosed ones who were not on antihypertensive treatments. The treatment was also considered safe with the safety outcome (SGA) remaining insignificant between the two studied groups (RR [95% CI] = 1.04 [0.82; 1.31]) and the rates of SGA were similar to the results of our analysis (CHAP study: 11.2% vs. 10.4% and our results: 14% vs. 13%).^6^ On the other hand, the CHIPS trial was another multicenter open‐label trial that was designed to assess whether or not, a tight blood pressure control (target DBP of 85 mm Hg) can yield better outcomes compared to a less‐tight approach (target DBP of 100 mm Hg) in pregnant women with gestational or chronic hypertension (DBP of 90–105 or 85–105 if already on pharmacologic treatment). The two groups did not differ in terms of primary outcomes (pregnancy loss, SGA, and high‐level neonatal care) and maternal complications although less‐tight control had significantly higher odds of developing severe hypertension. Although this study comprised both cases of chronic and gestational hypertension, no subgroup analysis was performed to investigate if there was a difference in results based on the type of hypertension. Also, many patients were already on antihypertensive treatment when enrolled in the study and remained so during the course of follow‐up, and no comparisons were made between patients already using treatment and patients whose medications were started for them during the course of the study. Thus, the findings of this trial could not confirm the risks or benefits of tight blood pressure control in pregnant women with hypertension.^5^ The statement made by SMFM in 2015 following the publication of the CHIPS results proposed skepticism in the treatment of mild to moderate chronic hypertension during pregnancy and they stated that patients diagnosed with mild to moderate chronic hypertension may need to discontinue using antihypertensives unless in case of severe hypertension.^24^ The latest statement published by the SMFM committee expressed that with the evidence provided by the results of the CHAP trial, this society supports lowering the blood pressure goal to less than 140/90 mm Hg in hypertensive patients during pregnancy.^7^ Our findings, which is the first meta‐analysis in the literature including the results of the CHAP trial, also confirm that the benefits of treatment of mild hypertension during pregnancy outweigh the risks since we found that treatment of mild cases of hypertension not only decreases the risks of adverse maternal outcomes, it also does not cause significant side effects.

Recently published meta‐analyses^25^, ^26^ have been focusing on the same issue with one of them including the results of only eight randomized studies and another one recruiting broader eligibility criteria by including trials comparing active treatment versus placebo or more versus less intensive active treatment. The results of the latter study^26^ demonstrated that active treatment or tight control regimen increases the risk of SGA compared to no treatment or less‐tight control raising concerns regarding the justification to use antihypertensives. It is noteworthy that, unlike the mentioned study, our results just like similar studies, support the use of antihypertensive medications for mild hypertension since our findings did not show any increased chance of developing SGA in patients taking active treatment. The results of a previous comprehensive meta‐analysis^27^ displayed that similar to our study, antihypertensive treatment of mild to moderate hypertension regardless of the type of hypertension and presence of proteinuria appeared to be more effective than no treatment. No evidence of a significant effect of treatment was found on preventing preeclampsia or proteinuria in that meta‐analysis. We found that antihypertensive medications can prevent the occurrence of preeclampsia in mild hypertensive patients but not proteinuria. Contrary to the mentioned meta‐analysis, we evaluated the impact of the intervention on preeclampsia and proteinuria separately and this may explain the difference between the two studies. Another important finding in our analysis was that after removing a study with a high possibility of bias, there was a statistically significant association between the treatment of mild cases of hypertension during pregnancy and decreased risk of neonatal mortality. This beneficial effect was not found in similar studies and it may warrant future large‐scale clinical trials and meta‐analyses to focus on the effect of treatment on this outcome. In another meta‐analysis on patients with chronic hypertension during pregnancy, it was shown that antihypertensive therapy did not change the odds of pregnancy outcomes except SGA which five studies were included in the analysis (odds ratio [95% CI] = 1.86 [1.38; 2.50]).^28^ The mentioned meta‐analysis did not stratify patients based on the severity of their hypertension and only included observational studies. Furthermore, the included studies for comparison of treatment and no treatment were limited. Given the high possibility of confounding factors in observational studies, the results of a meta‐analysis of RCTs with a larger sample size may yield more reliable results. A network meta‐analysis assessed the first‐line antihypertensive choices between hydralazine, nifedipine, and labetalol for patients with severe hypertension in pregnancy and demonstrated that although medications were not different regarding their impact on the incidence rate of cesarean delivery and maternal adverse effects, nifedipine appeared to be superior to hydralazine in the successful treatment of severe hypertension. The authors concluded that treatment with oral nifedipine as the first line of therapy in cases with severe hypertension during pregnancy shows a higher level of success in managing hypertension with the lowest episodes of hypotension.^29^ The majority of the studies included in our meta‐analysis used either nifedipine, labetalol, hydralazine, or methyldopa as the intervention arm in cases with mild to moderate hypertension but it is not clarified which choice of medication can outperform the others. In a recent network meta‐analysis, the first‐line treatments were compared regarding their impact on pregnancy outcomes in randomized trials of mild to moderate hypertension and the results yielded that although all the commonly prescribed antihypertensives can prevent severe hypertension, labetalol may also decrease the chances of proteinuria and perinatal death, unlike the other options.^30^

There were some strengths and limitations in this meta‐analysis. This is the first meta‐analysis to include the results of the recently published CHAP trial. Contrary to some of the previous meta‐analyses, we only included the results of the RCTs and hence, our results represent the best evidence and are less likely to be influenced by confounding factors. We assessed the impact of treatment on participants with chronic and pregnancy‐induced hypertension and the analyses were stratified based on their type of hypertension. Thus, the results can be used for both types of hypertension during pregnancy. Since the trials used different therapies as their intervention arm, this may impact the final results. The results of the meta‐analysis of proportions may not be reliable in analyses with a limited number of studies included. Since we included trials with both chronic and gestational hypertension, to decrease the impact of confounding variables, all the analyses were stratified based on the type of hypertensive patients included in each trial, but since there was a small number of trials in each subgroup, the results of the subgroup analyses may not be fully reliable and they should be interpreted with caution. Also, since the sample size of most included trials was lower than that of the CHAP trial, our results are highly influenced by the findings of this trial and this effect cannot be omitted.

## 5 CONCLUSION

Taken together, the results of the present meta‐analysis confirm the beneficial effects of antihypertensive treatment in patients with mild hypertension on pregnancy outcomes. Pharmacological treatment of mild hypertension during pregnancy contributes to lower risks of severe hypertension, preeclampsia, placental abruption, renal impairment, and pulmonary edema. The use of antihypertensive medications for patients diagnosed with mild hypertension appears to be also safe and it poses no higher risks of adverse pregnancy outcomes including SGA, LBW, and neonatal and fetal mortality. We may recommend that the adoption of lowering the favorite target of starting antihypertensive medications during pregnancy to the level of 140/90 mm Hg in patients affected by chronic and gestational hypertension in pregnancy seems acceptable.

## AUTHOR CONTRIBUTIONS

Armin Attar contributed to the conceptualization. Alireza Hosseinpour and Mana Moghadami screened the databases for eligible studies. Alireza Hosseinpour performed the data synthesis. Armin Attar and Alireza Hosseinpour took part in preparing the first draft and editing. All the authors read the submitted version of the manuscript and approved it.

## CONFLICT OF INTEREST STATEMENT

The authors declare no conflict of interest.

## Supporting information

Supplementary information.

## Data Availability

The data underlying this article will be shared on reasonable request from the corresponding author.
