# Supplementary material for: The impact of antihypertensive treatment of mild to moderate hypertension during pregnancy on maternal and neonatal outcomes: An updated meta‐analysis of randomized controlled trials
Source: Clin Cardiol. 2023 Mar 28;46(5):467–76. doi: 10.1002/clc.24013 (PMC10189071; doi:10.1002/clc.24013)
Supplement: Supplementary file 1 — Supplementary information. [file CLC-46-467-s001.docx]

**Keywords search strategy:**

A) PubMed

("antihypertensive agents"[Pharmacological Action] OR "antihypertensive agents"[MeSH Terms] OR ("antihypertensive"[All Fields] AND "agents"[All Fields]) OR "antihypertensive agents"[All Fields] OR ("anti"[All Fields] AND "hypertensive"[All Fields]) OR "anti hypertensive"[All Fields] OR ("antihypertensive agents"[Pharmacological Action] OR "antihypertensive agents"[MeSH Terms] OR ("antihypertensive"[All Fields] AND "agents"[All Fields]) OR "antihypertensive agents"[All Fields] OR ("anti"[All Fields] AND "hypertensive"[All Fields]) OR "anti hypertensive"[All Fields]) OR "acei"[All Fields] OR ("adrenergic beta antagonists"[Pharmacological Action] OR "adrenergic beta antagonists"[MeSH Terms] OR ("adrenergic"[All Fields] AND "beta antagonists"[All Fields]) OR "adrenergic beta antagonists"[All Fields] OR ("beta"[All Fields] AND "blocker"[All Fields]) OR "beta blocker"[All Fields]) OR ("antihypertension"[All Fields] OR "antihypertensive agents"[Pharmacological Action] OR "antihypertensive agents"[MeSH Terms] OR ("antihypertensive"[All Fields] AND "agents"[All Fields]) OR "antihypertensive agents"[All Fields] OR "antihypertensive"[All Fields] OR "antihypertensives"[All Fields]) OR ("diuretics"[Pharmacological Action] OR "diuretics"[MeSH Terms] OR "diuretics"[All Fields] OR "diuretic"[All Fields]) OR ("thiazid"[All Fields] OR "thiazides"[MeSH Terms] OR "thiazides"[All Fields] OR "thiazide"[All Fields] OR "thiazidic"[All Fields]) OR ("calcium channel blockers"[Pharmacological Action] OR "calcium channel blockers"[MeSH Terms] OR ("calcium"[All Fields] AND "channel"[All Fields] AND "blockers"[All Fields]) OR "calcium channel blockers"[All Fields] OR ("calcium"[All Fields] AND "channel"[All Fields] AND "blocker"[All Fields]) OR "calcium channel blocker"[All Fields]) OR ("angiotensin receptor antagonists"[Pharmacological Action] OR "angiotensin receptor antagonists"[MeSH Terms] OR ("angiotensin"[All Fields] AND "receptor"[All Fields] AND "antagonists"[All Fields]) OR "angiotensin receptor antagonists"[All Fields] OR ("angiotensin"[All Fields] AND "receptor"[All Fields] AND "blocker"[All Fields]) OR "angiotensin receptor blocker"[All Fields]) OR ("peptidyl dipeptidase a"[MeSH Terms] OR "peptidyl dipeptidase a"[All Fields] OR ("angiotensin"[All Fields] AND "converting"[All Fields] AND "enzyme"[All Fields]) OR "angiotensin converting enzyme"[All Fields]) OR "arb"[All Fields] OR ("hydralazine"[MeSH Terms] OR "hydralazine"[All Fields]) OR ("labetalol"[MeSH Terms] OR "labetalol"[All Fields]) OR ("methyldopa"[MeSH Terms] OR "methyldopa"[All Fields]) OR ("therapeutics"[MeSH Terms] OR "therapeutics"[All Fields] OR "treatments"[All Fields] OR "therapy"[MeSH Subheading] OR "therapy"[All Fields] OR "treatment"[All Fields] OR "treatment s"[All Fields])) AND ("pre eclampsia"[MeSH Terms] OR "pre eclampsia"[All Fields] OR "preeclampsia"[All Fields] OR ("premature birth"[MeSH Terms] OR ("premature"[All Fields] AND "birth"[All Fields]) OR "premature birth"[All Fields] OR ("preterm"[All Fields] AND "birth"[All Fields]) OR "preterm birth"[All Fields]) OR ("abruptio placentae"[MeSH Terms] OR ("abruptio"[All Fields] AND "placentae"[All Fields]) OR "abruptio placentae"[All Fields] OR ("placental"[All Fields] AND "abruption"[All Fields]) OR "placental abruption"[All Fields]) OR ("foetal death"[All Fields] OR "fetal death"[MeSH Terms] OR ("fetal"[All Fields] AND "death"[All Fields]) OR "fetal death"[All Fields]) OR ("perinatal death"[MeSH Terms] OR ("perinatal"[All Fields] AND "death"[All Fields]) OR "perinatal death"[All Fields] OR ("neonatal"[All Fields] AND "death"[All Fields]) OR "neonatal death"[All Fields]) OR (("small"[Journal] OR "small"[All Fields]) AND ("gestational age"[MeSH Terms] OR ("gestational"[All Fields] AND "age"[All Fields]) OR "gestational age"[All Fields])) OR (("cardiovascular system"[MeSH Terms] OR ("cardiovascular"[All Fields] AND "system"[All Fields]) OR "cardiovascular system"[All Fields] OR "cardiovascular"[All Fields] OR "cardiovasculars"[All Fields]) AND ("complicances"[All Fields] OR "complicate"[All Fields] OR "complicated"[All Fields] OR "complicates"[All Fields] OR "complicating"[All Fields] OR "complication"[All Fields] OR "complication s"[All Fields] OR "complications"[MeSH Subheading] OR "complications"[All Fields])) OR ("maternal death"[MeSH Terms] OR ("maternal"[All Fields] AND "death"[All Fields]) OR "maternal death"[All Fields]) OR ("maternal mortality"[MeSH Terms] OR ("maternal"[All Fields] AND "mortality"[All Fields]) OR "maternal mortality"[All Fields]) OR ("heart failure"[MeSH Terms] OR ("heart"[All Fields] AND "failure"[All Fields]) OR "heart failure"[All Fields]) OR ("stroke"[MeSH Terms] OR "stroke"[All Fields] OR "strokes"[All Fields] OR "stroke s"[All Fields]) OR ("myocardial infarction"[MeSH Terms] OR ("myocardial"[All Fields] AND "infarction"[All Fields]) OR "myocardial infarction"[All Fields]) OR ("pulmonary oedema"[All Fields] OR "pulmonary edema"[MeSH Terms] OR ("pulmonary"[All Fields] AND "edema"[All Fields]) OR "pulmonary edema"[All Fields]) OR ("eclampsia"[MeSH Terms] OR "eclampsia"[All Fields] OR "eclampsias"[All Fields]) OR ("hellp syndrome"[MeSH Terms] OR ("hellp"[All Fields] AND "syndrome"[All Fields]) OR "hellp syndrome"[All Fields] OR "hellp"[All Fields]) OR ("end"[All Fields] AND ("j organ dysfunct"[Journal] OR ("organ"[All Fields] AND "dysfunction"[All Fields]) OR "organ dysfunction"[All Fields])) OR ("caesarean section"[All Fields] OR "cesarean section"[MeSH Terms] OR ("cesarean"[All Fields] AND "section"[All Fields]) OR "cesarean section"[All Fields]) OR ("cesarean section"[MeSH Terms] OR ("cesarean"[All Fields] AND "section"[All Fields]) OR "cesarean section"[All Fields] OR ("cesarean"[All Fields] AND "delivery"[All Fields]) OR "cesarean delivery"[All Fields]) OR ("abortion, spontaneous"[MeSH Terms] OR ("abortion"[All Fields] AND "spontaneous"[All Fields]) OR "spontaneous abortion"[All Fields] OR "miscarriage"[All Fields] OR "miscarriages"[All Fields]) OR ("stillbirth"[MeSH Terms] OR "stillbirth"[All Fields] OR ("still"[All Fields] AND "birth"[All Fields]) OR "still birth"[All Fields]) OR ("infant, low birth weight"[MeSH Terms] OR ("infant"[All Fields] AND "low"[All Fields] AND "birth"[All Fields] AND "weight"[All Fields]) OR "low birth weight infant"[All Fields] OR ("low"[All Fields] AND "birth"[All Fields] AND "weight"[All Fields]) OR "low birth weight"[All Fields])) AND (((("chronic"[All Fields] OR "chronical"[All Fields] OR "chronically"[All Fields] OR "chronicities"[All Fields] OR "chronicity"[All Fields] OR "chronicization"[All Fields] OR "chronics"[All Fields]) AND ("hypertense"[All Fields] OR "hypertension"[MeSH Terms] OR "hypertension"[All Fields] OR "hypertension s"[All Fields] OR "hypertensions"[All Fields] OR "hypertensive"[All Fields] OR "hypertensive s"[All Fields] OR "hypertensives"[All Fields])) OR ("hypertension, pregnancy induced"[MeSH Terms] OR ("hypertension"[All Fields] AND "pregnancy induced"[All Fields]) OR "pregnancy-induced hypertension"[All Fields] OR ("gestational"[All Fields] AND "hypertension"[All Fields]) OR "gestational hypertension"[All Fields])) AND ("pregnancy"[MeSH Terms] OR "pregnancy"[All Fields] OR "pregnancies"[All Fields] OR "pregnancy s"[All Fields]))

B) Scopus

( ( TITLE-ABS-KEY ( anti-hypertensive ) OR TITLE-ABS-KEY ( anti AND hypertensive ) OR TITLE-ABS-KEY ( acei ) OR TITLE-ABS-KEY ( beta AND blocker ) OR TITLE-ABS-KEY ( antihypertensive ) OR TITLE-ABS-KEY ( diuretic ) OR TITLE-ABS-KEY ( thiazide ) OR TITLE-ABS-KEY ( calcium AND channel AND blocker ) OR TITLE-ABS-KEY ( angiotensin AND receptor AND blocker ) OR TITLE-ABS-KEY ( angiotensin AND converting AND enzyme ) OR TITLE-ABS-KEY ( arb ) OR TITLE-ABS-KEY ( hydralazine ) OR TITLE-ABS-KEY ( methyldopa ) OR TITLE-ABS-KEY ( treatment ) ) ) AND ( ( TITLE-ABS-KEY ( chronic AND hypertension ) OR TITLE-ABS-KEY ( gestational AND hypertension ) AND TITLE-ABS-KEY ( pregnancy ) ) ) AND ( ( TITLE-ABS-KEY ( preeclampsia ) OR TITLE-ABS-KEY ( preterm AND birth ) OR TITLE-ABS-KEY ( placental AND abruption ) OR TITLE-ABS-KEY ( fetal AND death ) OR TITLE-ABS-KEY ( neonatal AND death ) OR TITLE-ABS-KEY ( small AND for AND gestational AND age ) OR TITLE-ABS-KEY ( cardiovascular AND complication ) OR TITLE-ABS-KEY ( maternal AND death ) OR TITLE-ABS-KEY ( maternal AND mortality ) OR TITLE-ABS-KEY ( heart AND failure ) OR TITLE-ABS-KEY ( stroke ) OR TITLE-ABS-KEY ( myocardial AND infarction ) OR TITLE-ABS-KEY ( pulmonary AND edema ) OR TITLE-ABS-KEY ( eclampsia ) OR TITLE-ABS-KEY ( hellp ) OR TITLE-ABS-KEY ( end AND organ AND dysfunction ) OR TITLE-ABS-KEY ( cesarean ) OR TITLE-ABS-KEY ( miscarriage ) OR TITLE-ABS-KEY ( stillbirth ) OR TITLE-ABS-KEY ( low AND birth AND weight ) ) )

C) Embase

(('chronic hypertension'/exp OR 'chronic hypertension' OR (chronic AND ('hypertension'/exp OR hypertension))) OR (' gestational hypertension'/exp OR gestational hypertension)) AND ('pregnancy'/exp OR pregnancy) AND (preeclampsia:ti,ab,kw OR 'preterm birth':ti,ab,kw OR 'placental abruption':ti,ab,kw OR 'fetal death':ti,ab,kw OR 'neonatal death':ti,ab,kw OR 'neonatal mortality':ti,ab,kw OR 'small for gestational age':ti,ab,kw OR 'cardiovascular complication':ti,ab,kw OR 'maternal death':ti,ab,kw OR 'maternal mortality':ti,ab,kw OR 'heart failure':ti,ab,kw OR stroke:ti,ab,kw OR 'myocardial infarction':ti,ab,kw OR 'pulmonary edema':ti,ab,kw OR eclampsia:ti,ab,kw OR hellp:ti,ab,kw OR 'end organ dysfunction':ti,ab,kw OR 'cesarean section':ti,ab,kw OR 'cesarean delivery':ti,ab,kw OR miscarriage:ti,ab,kw OR stillbirth:ti,ab,kw OR 'low birth weight':ti,ab,kw) AND ('anti hypertensive':ti,ab,kw OR acei:ti,ab,kw OR 'beta blocker':ti,ab,kw OR antihypertensive:ti,ab,kw OR diuretic:ti,ab,kw OR thiazide:ti,ab,kw OR 'calcium channel blocker':ti,ab,kw OR 'angiotensin receptor blocker':ti,ab,kw OR 'angiotensin converting enzyme':ti,ab,kw OR arb:ti,ab,kw OR hydralazine:ti,ab,kw OR methyldopa:ti,ab,kw OR treatment:ti,ab,kw OR labetalol:ti,ab,kw)

| **Pregnancy outcomes** | **Definitions** |
| --- | --- |
| Severe hypertension | Systolic blood pressure ≥ 160 mm Hg or diastolic blood pressure ≥ 110 mm Hg |
| Preeclampsia | New onset proteinuria with at least 300 mg protein in a 24 hour urine sample after 20 weeks of gestation |
| Preterm delivery | Delivery before 37 weeks of gestation |
| Placental abruption | Complete or partial separation of placenta from the uterine wall before birth |
| ECG change | Strain pattern present in electrocardiogram |
| Hepatic impairment | Elevated liver enzymes twice the normal limit |
| Renal impairment | Elevated serum creatinine > 1.1 mg/dl |
| SGA | Birth weight at the lower extreme of the normal birth weight distribution (usually < 10^th^ percentile for gestational age which can be due to different etiologies) |
| IUFD | Fetal death at or after 20 weeks of gestation |
| LBW | Neonatal weight < 2500 g |
| Apgar < 7 | Apgar score <7 after 5 minutes of birth |
| Neonatal mortality | Neonatal death within the first four weeks of delivery |

**Table S1:** definition of outcomes of interest (ECG: electrocardiogram, SGA: small for gestational age, IUFD: intrauterine fetal demise, and LBW: low birth weight)


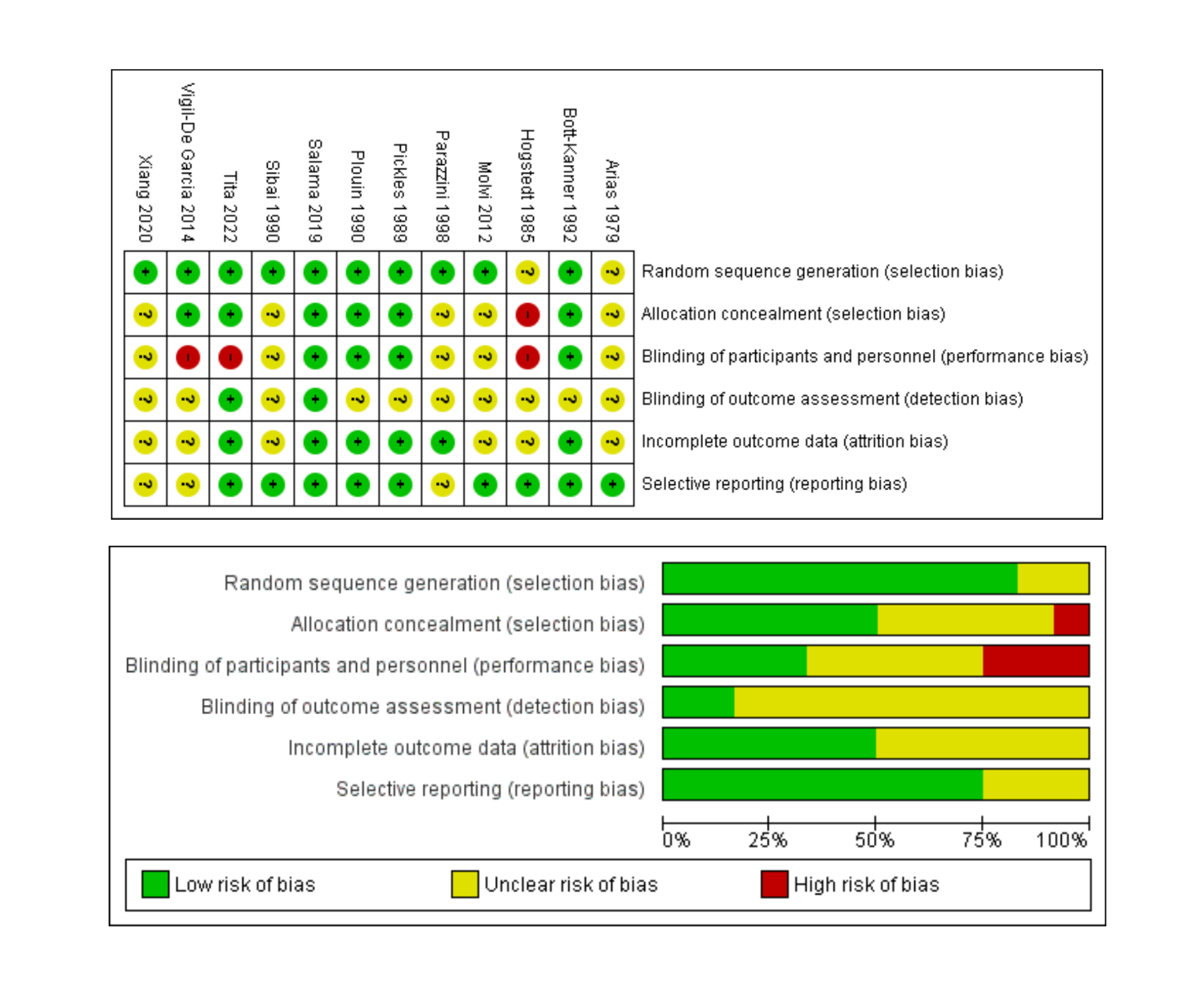


**Figure S1:** Risk of bias graph and summary

**
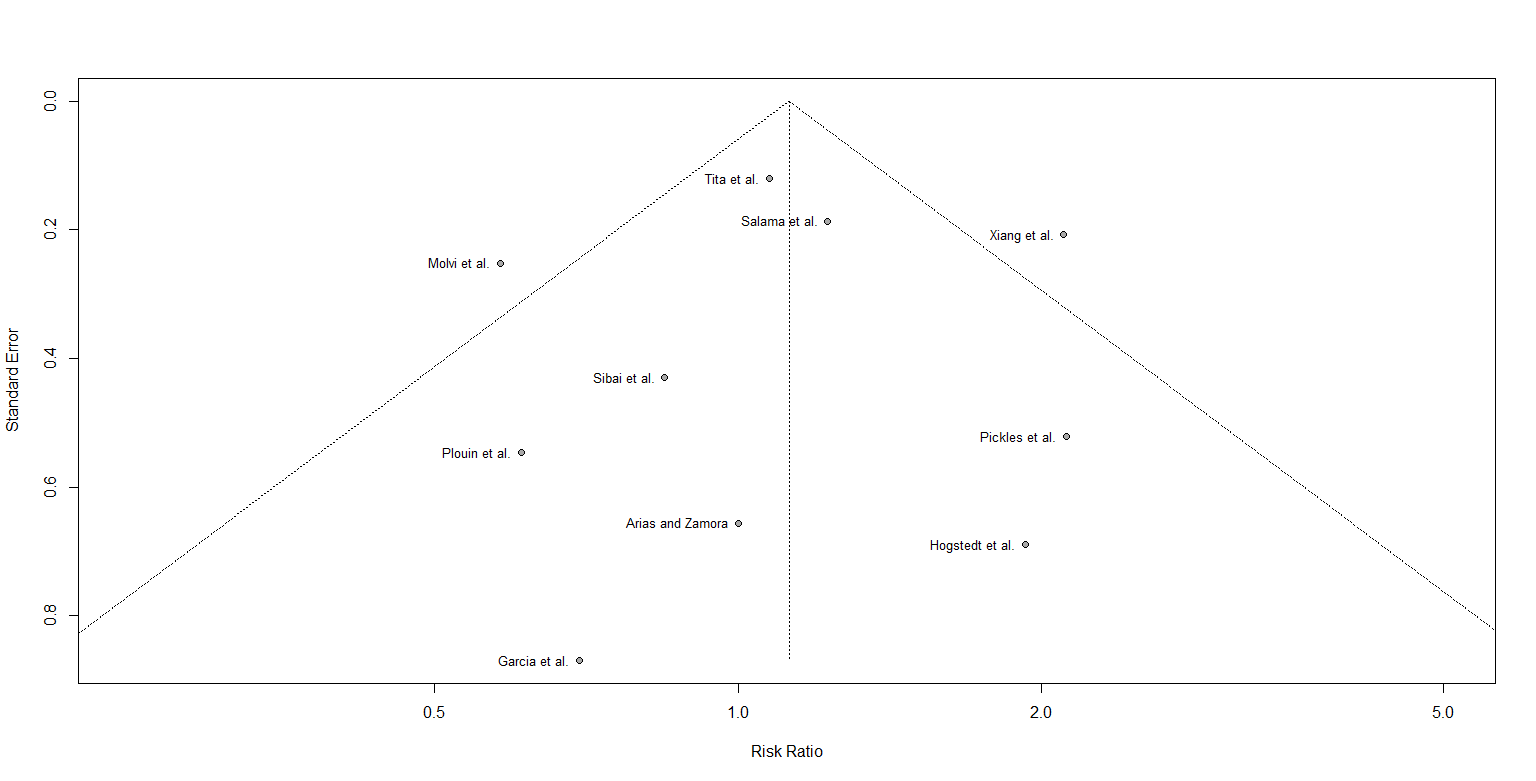
**

**Figure S2:** Funnel plot showing distribution of studies based on the safety outcome (small for gestational age)


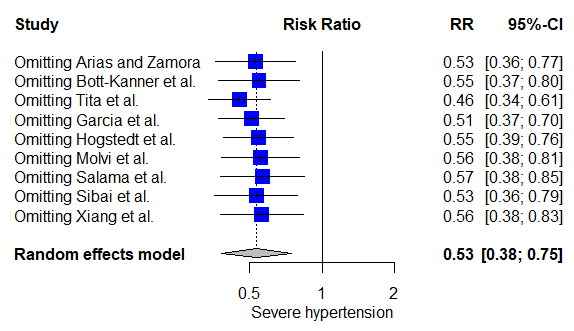


**Figure S3:** Sensitivity analysis of risk ratio of severe hypertension (RR: risk ratio, 95%-CI: 95% confidence interval)


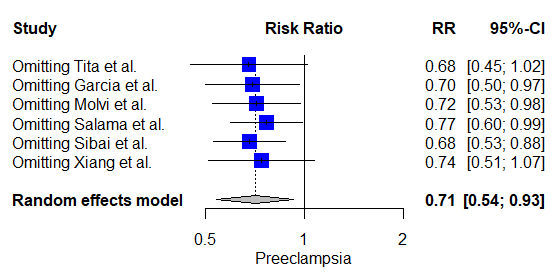


**Figure S4:** Sensitivity analysis of risk ratio of preeclampsia (RR: risk ratio, 95%-CI: 95% confidence interval)

**
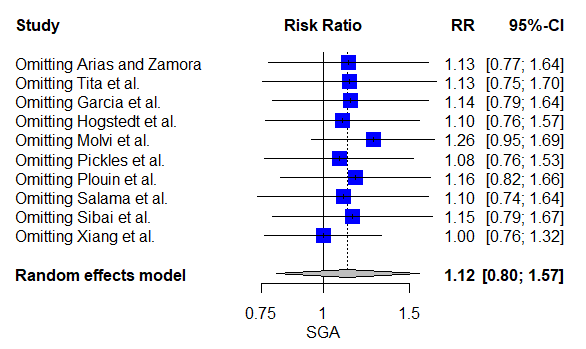
**

**Figure S5:** Sensitivity analysis of risk ratio of small for gestational age (RR: risk ratio, 95%-CI: 95% confidence interval, SGA: small for gestational age)

**
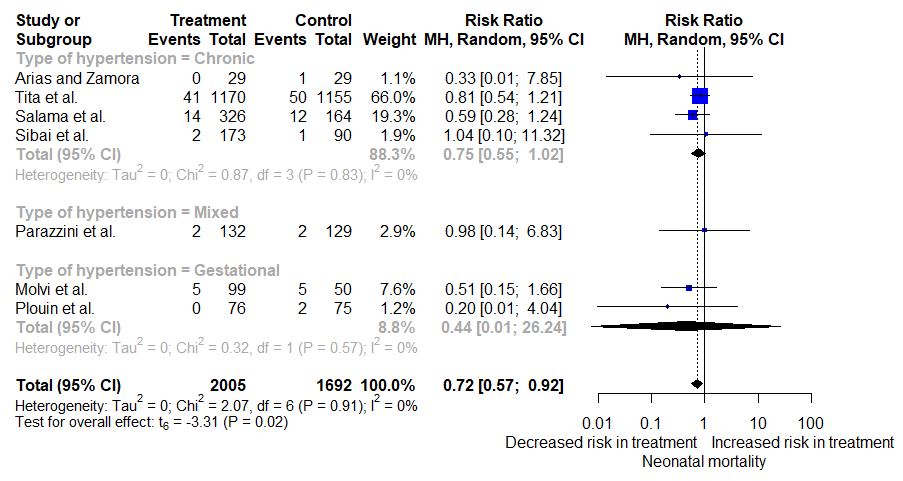
**

**Figure S6:** Forrest plot displaying the impact of antihypertensive treatment on neonatal mortality compared to the control group receiving no treatment/placebo after exclusion of the study with a potential risk of bias (MH: Mantel-Haenszel, 95% CI: 95% confidence interval)

**
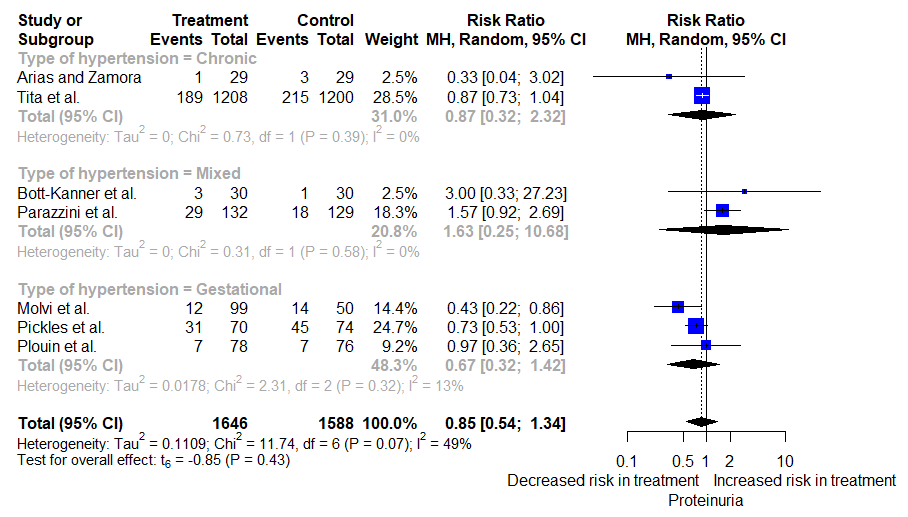
**

**Figure S7:** Forest plot of association between antihypertensive treatment of mild hypertension and proteinuria during pregnancy (MH: Mantel-Haenszel, 95% CI: 95% confidence interval)

**
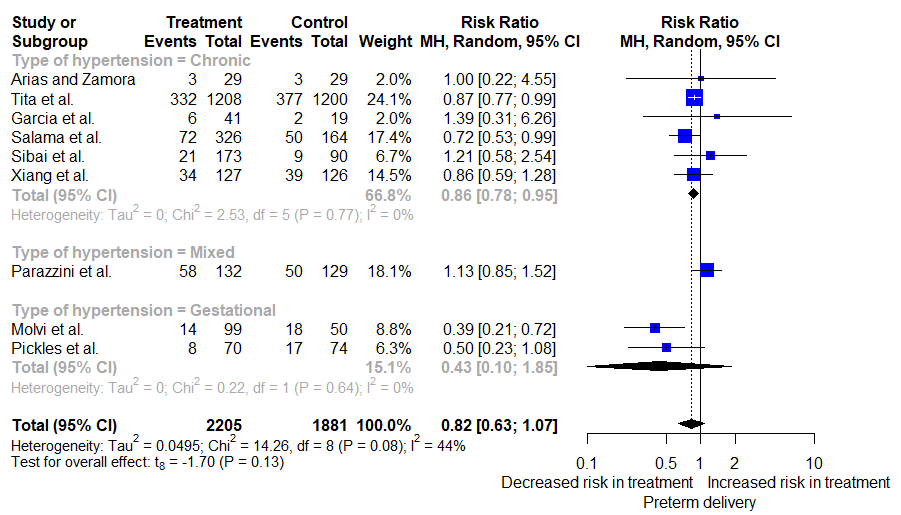
**

**Figure S8:** Forest plot of association between antihypertensive treatment of mild hypertension and preterm delivery during pregnancy (MH: Mantel-Haenszel, 95% CI: 95% confidence interval)

**
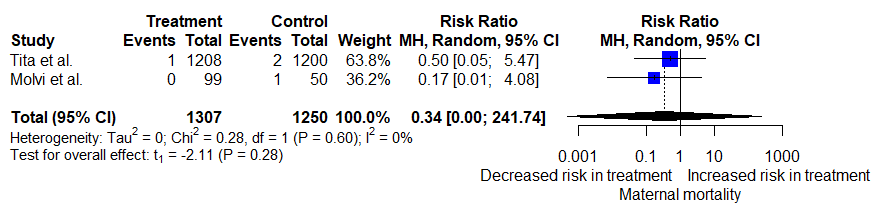
**

**Figure S9:** Forest plot of association between antihypertensive treatment of mild hypertension and maternal mortality during pregnancy (MH: Mantel-Haenszel, 95% CI: 95% confidence interval)

**
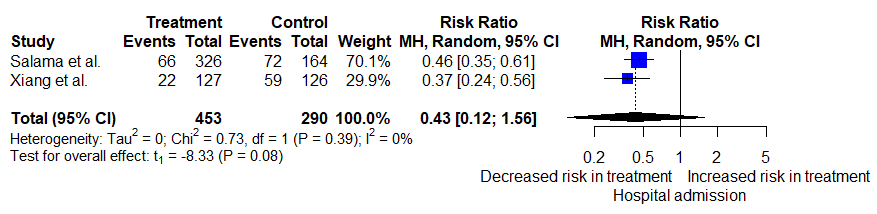
**

**Figure S10:** Forest plot of association between antihypertensive treatment of mild hypertension and hospital admission during pregnancy (MH: Mantel-Haenszel, 95% CI: 95% confidence interval)

**
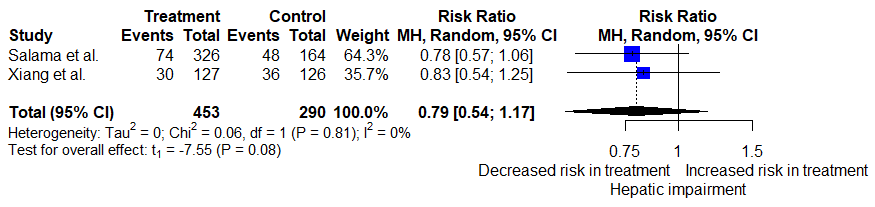
**

**Figure S11:** Forest plot of association between antihypertensive treatment of mild hypertension and hepatic impairment during pregnancy (MH: Mantel-Haenszel, 95% CI: 95% confidence interval)

**
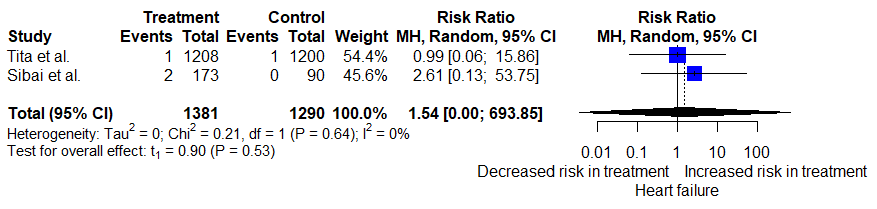
**

**Figure S12:** Forest plot of association between antihypertensive treatment of mild hypertension and heart failure during pregnancy (MH: Mantel-Haenszel, 95% CI: 95% confidence interval)

**
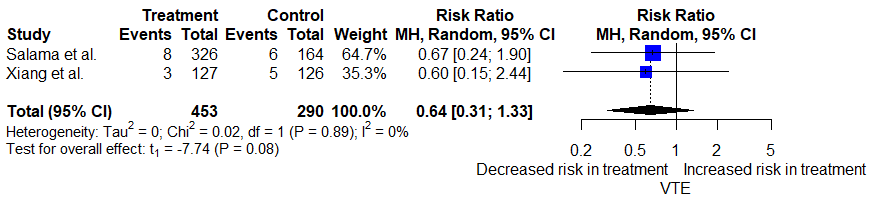
**

**Figure S13:** Forest plot of association between antihypertensive treatment of mild hypertension and venous thromboembolism during pregnancy (VTE: venous thromboembolism, MH: Mantel-Haenszel, 95% CI: 95% confidence interval)

**
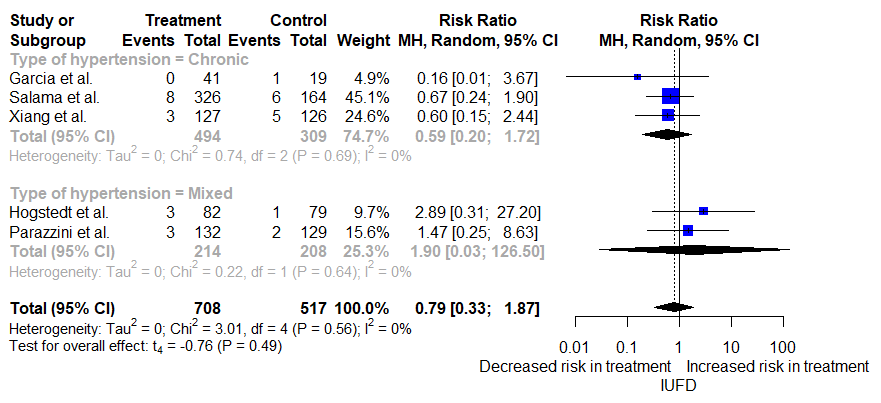
**

**Figure S14:** Forest plot of association between antihypertensive treatment of mild hypertension and IUFD during pregnancy (IUFD: intrauterine fetal demise, MH: Mantel-Haenszel, 95% CI: 95% confidence interval)

**
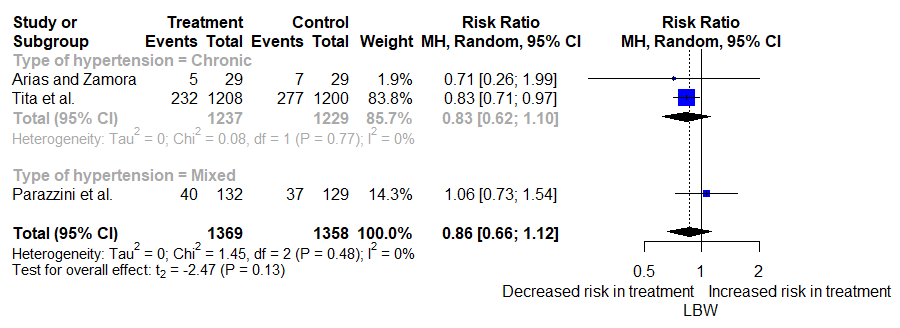
**

**Figure S15:** Forest plot of association between antihypertensive treatment of mild hypertension and low birth weight during pregnancy (LBW: low birth weight, MH: Mantel-Haenszel, 95% CI: 95% confidence interval)

**
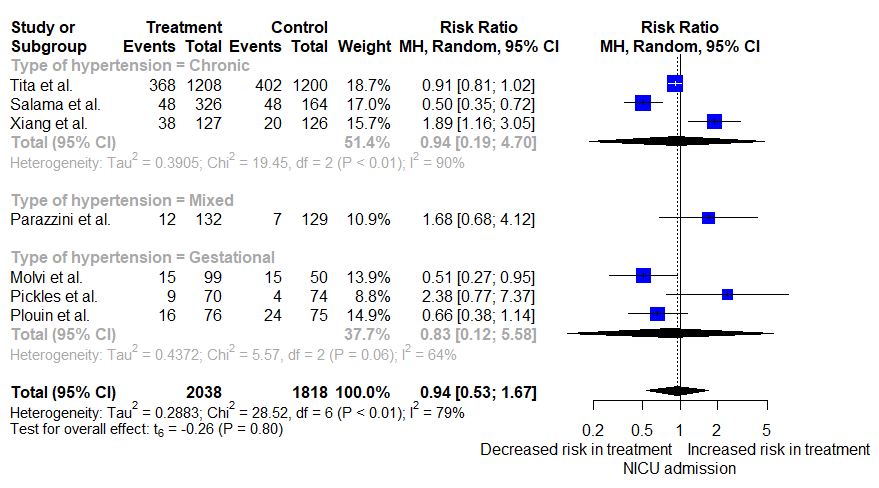
**

**Figure S16:** Forest plot of association between antihypertensive treatment of mild hypertension and neonatal intensive care unit during pregnancy (NICU: neonatal intensive care unit, MH: Mantel-Haenszel, 95% CI: 95% confidence interval)

**
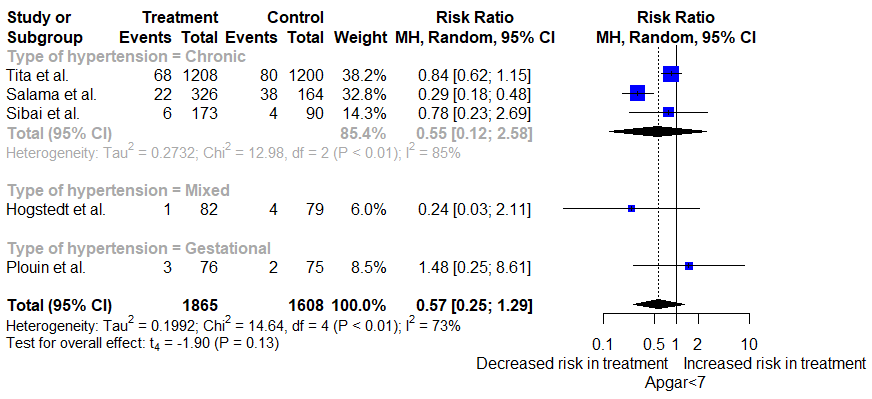
**

**Figure S17:** Forest plot of association between antihypertensive treatment of mild hypertension and Apgar score < 7 during pregnancy (MH: Mantel-Haenszel, 95% CI: 95% confidence interval)

**
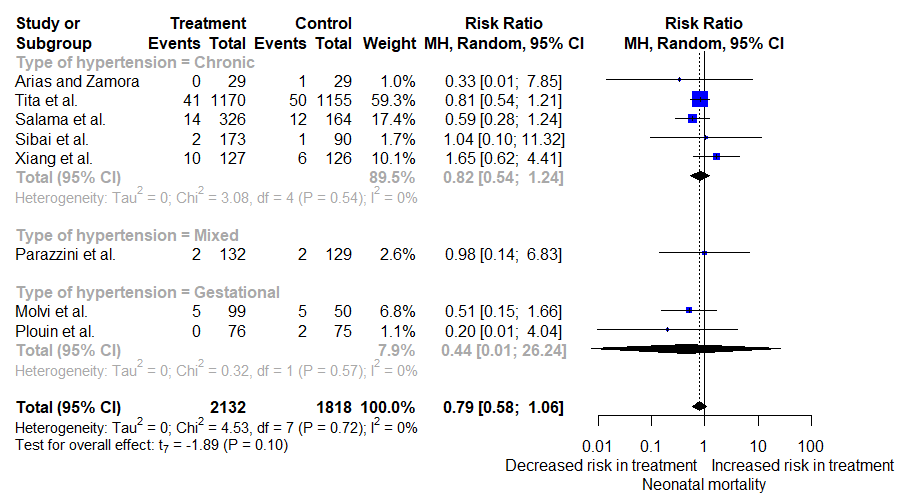
**

**Figure S18:** Forest plot of association between antihypertensive treatment of mild hypertension and neonatal mortality during pregnancy (MH: Mantel-Haenszel, 95% CI: 95% confidence interval)
